# Molecular modelling of fullerene C_60_ functionalized by nitric oxide for use in biological environment

**DOI:** 10.1038/s41598-024-53050-y

**Published:** 2024-01-31

**Authors:** Omid Moztarzadeh, Morteza Jamshidi, Avat Arman Taherpour, Vaclav Babuska

**Affiliations:** 1 Department of Stomatology, University Hospital Pilsen, Faculty of Medicine in Pilsen, Charles University, 323 00 Pilsen, Czech Republic; 2 Department of Anatomy, Faculty of Medicine in Pilsen, Charles University, 323 00 Pilsen, Czech Republic; 3 Young Researchers and Elite Club, Kermanshah Branch, Islamic Azad University, Kermanshah, Iran; 4 Chemistry Department, Faculty of Chemistry, Razi University, Kermanshah, Iran; 5 Department of Medical Chemistry and Biochemistry, Faculty of Medicine in Pilsen, Charles University, 323 00 Pilsen, Czech Republic

**Keywords:** Biomaterials - cells, Drug delivery, Computational chemistry

## Abstract

The unique potential of fullerene C_60_ for various biological applications has ignited significant interest. However, its inherent non-polarity poses a critical challenge for its effective integration within biological systems. This study delves into the intricate physicochemical characteristics of the innovative [C_60_ + NO] complex using density functional theory and time-dependent density functional theory. The computational analyses encompass molecular charge, surface electrostatic potential, and dipole moment evaluations. Impressively, the dipole moment of the [C_60_ + NO] complex significantly increases to 12.92 D. Meticulous surface analysis reveals a subtle interplay between molecular structures, indicating weak interactions. The analysis of the absorption spectrum unveils a noteworthy red-shift of 200 nm subsequent to complex formation. To elucidate the electron transfer mechanisms, we explore photo-induced electron transfer through CAM-B3LYP. This exploration elucidates intricate pathways governing electron transfer, with complementary insights gleaned from Marcus theory's outputs, especially the Gibbs free energy of electron transfer. Changes in the physicochemical properties of approaching C_60_ and NO molecules reveal interesting results compared to separate molecules. These findings resonate profoundly in the context of potential biological and pharmaceutical utilization. With implications for the biomedical area, the outcomes linked to the [C60 + NO] complex kindle optimism for pioneering biomedical applications.

## Introduction

Fullerenes have attracted significant attention due to their interesting properties and potential applications in various fields, including materials science, chemistry, and medicine. Although fullerene C_60_ is generally considered to be insoluble in water under normal conditions^1^, its solubility can be modified by chemical functionalization or other techniques to improve its dispersibility or solubility in aqueous systems. To enhance the solubility, the C_60_ molecule can be conjugated with certain groups and molecules^2^. There are several methods, such as chemical, electrochemical, photochemical, and biological, to introduce various functional groups such as hydroxyl^3^, carboxyl^4^, amino^5^, thiol^6^, and others onto the fullerene surface. These groups can be further conjugated with substances such as amino acids, peptides, sugars or even DNA. The attachment to the surface can impart specific properties or functionalities that can enable the interaction with biological systems. In recent decades, fullerene conjugates have become an important area of research for biomedical applications involving drug delivery^7,8^, antioxidant activity^9^, photodynamic therapy^10^, biological imaging^11^, or smart biomaterials in tissue engineering^12,13^.

There are various methods for the synthesis of fullerene derivatives due to the electron properties and its integrated π system^14–16^. In addition to the fullerene derivatives, the formation of its complex with some organic compounds, including drugs and vitamins^17^, increases the solubility of fullerene in water, which can be attributed to the high performance of the C_60_ Fullerene-Doxorubicin complex in the treatment of cancer^18,19^. This approach, in addition to changing the polarity of fullerenes, is also widely used as a drug carrier for blood–brain barrier penetration^20^. The production of fullerene colloids is also one of the other methods to increase the solubility of fullerenes in water. In this method, which is made possible by changing the solvent, ethanol is used as the human body limit^21^.

Although research data already exists for many conjugated fullerenes, there is a lack of information on the mechanism of the interaction between gases and fullerene derivatives^22,23^. An interesting compound with potential medical applications is that between fullerene C_60_ and nitric oxide.

Nitric oxide (NO) is ubiquitous in the environment and biological systems, playing important roles in physiological processes, cellular signaling, and medical applications. Its versatility and biological activities make it a molecule with widespread implications in various fields of research. In the environment, it forms a complex of nitrogen oxides (NO_x_) with an impact on air pollution and smog formation^24,25^. However, in the biological systems it is a signaling molecule playing a critical role in many physiological and pathological processes. In animals and humans, NO is synthesized by a family of enzymes called nitric oxide synthases^26^ and is involved in several physiological processes, including blood pressure regulation^27^, neurotransmission^28^, immune response against pathogens^29^, and inflammation^30^. Additionally, NO is also used in medical imaging techniques, such as positron emission tomography, where it is used as a radiotracer to study blood flow, metabolism, and other physiological processes in the body^31^. NO has also been used therapeutically in medical applications as a drug to treat certain cardiovascular conditions, such as persistent pulmonary hypertension of the newborn, which is a potentially life-threatening condition in which newborns have high blood pressure in the blood vessels of their lungs. In this condition, NO can help to relax the blood vessels in the lungs, improving blood flow and oxygenation^32^. Finally, nitroglycerin, which has been used for many decades to treat angina pectoris without knowing its effect, releases NO from its molecule^33^.

When C_60_ and NO react with each other, the NO molecule can be attached to the surface of the C_60_ molecule, forming a stable complex. This nitrosylated fullerene complex may have a number of interesting properties with potential applications in a variety of fields, including biomedicine and nanotechnology.

To better understand the properties of the [C_60_ + NO] complex formation, it is suitable to examine its excited state electron properties in more detail. Fullerene C_60_ plays a receptive role in the low energy LUMO orbital (about − 3 eV), along with electronegative compounds such as most conducting and semiconducting organic compounds with higher LUMO energy levels. However, a molecule such as NO can take charge due to the lower energy level of the LUMO orbital compared to the fullerene in the excited state. Consequently, as a result of the charge transfer (CT), there will be a significant change in the absorption optical spectrum and the elongation of the C_60_ fullerene molecule. Electron transfer and CT are among the most important chemical processes in nature, playing a central role in many biological, physical, and chemical systems (both organic and inorganic)^34^. Electronic solid states depend on the control of electron transfer in semiconductors, and the new environment of electronic molecules is crucial for understanding and controlling the transfer of electrons in and between molecules and nanostructures^35^. Another reason for studying the electron transfer process is that it is a very simple process of chemical reactions and is easy to understand. This phenomenon can be achieved in other types of chemical and biochemical reactions.

Different complexes of fullerene C_60_ have also been simulated with various computational methods along with drugs and some organic molecules^36^; so that they can be used as a suitable model for the study of other organic substances needed in the body. Using hydrophilic Docking C_60_ calculations showed that it has proper interactions (van der Waals) with proteins of several groups, including acetylcholinesterase, glutamate racemase, inosine monophosphate dehydrogenase, lumazine synthase, human estrogen receptor alpha, dihydrofolate reductase, and N-myristoyltransferase, and can be used as a carrier of reliable and effective drug. It can be used directly from some of its therapeutic properties^37^. Also, simulations based on Docking calculations have shown that the interaction of ligands such as dimethyl-N-(benzoyl)-amidophosphate (HCP) and dimethyl-N-(phenylsulfonyl)-amidophosphate (HSP) with DNA is much stronger than that of fullerene C_60_ with DNA, which itself indicates that fullerene has the ability to transfer drugs or ligands to the target and then has no ability to compete with them and leaves the target^38^. A notable example of the formulation of a nanocarrier system for drug delivery is the use of graphene as a carrier for both doxorubicin^39^, an effective anticancer drug, and amiodarone^40^, a cardiovascular drug. In another study, the adsorption of CO and NO molecules on pristine, Sc, Ti, V, Cr, Mn, Fe, Co, Ni, Cu and Zn doped fullerene C_60_ was theoretically investigated^41^. NO and NO_x_ have been identified in various DFT and TD-DFT studies conducted on different nanostructures, such as the C_6_N_8_ monolayer^42^.

According to our experience so far, this work is performed for the first time with the aim of studying the electron and optical properties of the [C_60_ + NO] complex. Based on this view that a donor–acceptor system is formed^43^, calculations and studies are carried out. These two molecules can be characterized by the formation of the complex [C_60_ + NO]. Using Density Functional Theory (DFT) and Time Dependent Density Functional Theory (TD-DFT) computational methods, some of the significant electron and optical properties are evaluated by a different approach using new parameters. Attempts are made to completely analyze the excitations in the excited state and, in addition to examining the optical absorption spectrum, the distributed spectra are calculated and the modes of load transitions between these two species are determined. Also, the electron transfer rate at the time of excitation and the electron lifetime at the time of distribution are obtained using the Marcus theory. A proper comprehension of the behavior of newly designed molecules is crucial to understanding their potential for use in biological systems and other applications.

## Computational methods

### Ground state optimization

**Figure 1:**
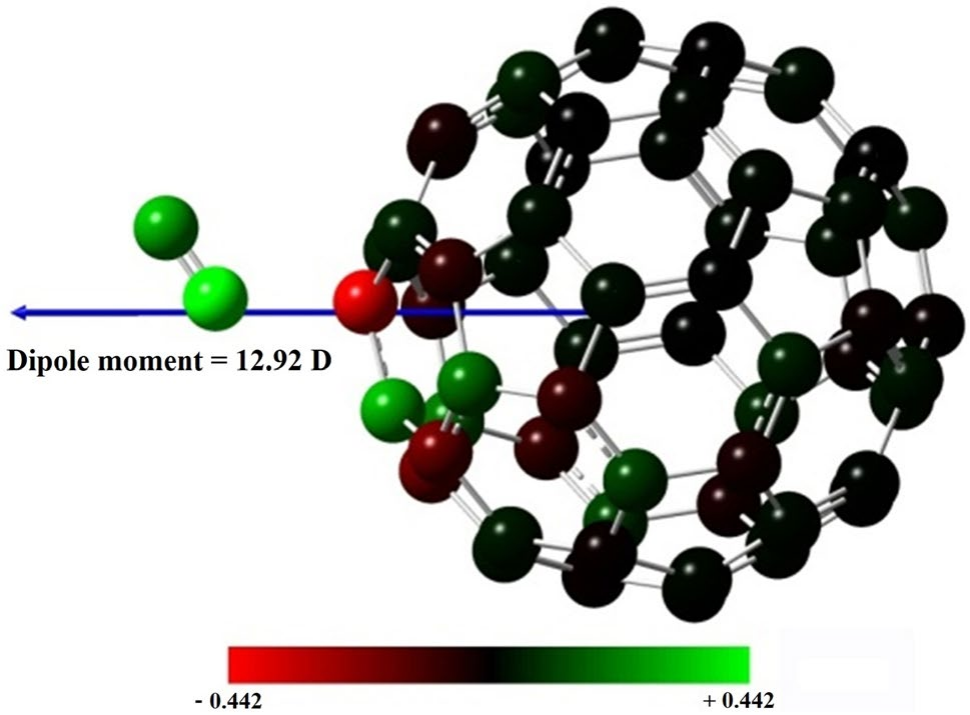
Dipole moment and complex Mulliken charge [C_60_ + NO] in the gas phase computed by B3LYP/6–31 + G*.

In this work, we considered only one complex of NO and C_60_ with ratios of one to one. The molecules of C_60_, NO, and the complex [C_60_ + NO] are optimized separately in the gas phase using the DFT B3LYP method^44^ and the base set of 6–31 + G*. The 6-31G* base set, applicable to most atoms up to third-row atoms, includes a proper approximation for organic and carbon structures. Additionally, it accurately calculates charge dispersion on surfaces, especially effective for spherical systems like fullerene. The basic functions, a broader application of the 6-31G and 6-31G* sets up to Ar, utilize six primitive Gaussians for 1s, 2s, 2p, 3s, and 3p. Furthermore, a split-valence pair of three and one primitives is employed for valence orbitals, ensuring comprehensive coverage for molecular structures^45–47^. The formation of the [C_60_ + NO] complex is accomplished with placing these two molecules at 3 Å from each other without the directional orientation of nitrogen or oxygen, which, after optimizing the NO gas closing from the oxygen head to the fullerene C_60_ and the desired complex in the equilibrium distance of 1.92 Å is formed according to the Fig. 1. The energy of the complex formation, based on the Gibbs free energy, is calculated through the vibrational frequency according to Eq. (1). ^48^

1 $${\text{E}}_{{{\text{Formation}}}} = {\text{E}}_{{{\text{complex}}}} {-}{\text{E}}_{{{\text{ingredients}}}}$$

Through Eqs. (2–6), with the use of the energy of HOMO or LUMO orbitals, the values of electronegativity (*X*), hardness (*η*), softness (*S*), electrophysical (*ω*) and chemical potentials (*μ*) are obtained, respectively. The calculated results are in Table 1.

2 $${\text{X }} = -\upmu$$

3 $$\upmu = \left( {1/2} \right)\left( {{\text{E}}_{{{\text{HOMO}}}} + {\text{E}}_{{{\text{LUMO}}}} } \right)$$

4 $$\upeta = \left( {{1}/{2}} \right)\left( {{\text{E}}_{{{\text{LUMO}}}} - {\text{E}}_{{{\text{HOMO}}}} } \right)$$

5 $${\text{S}} = {1/}\upeta$$

6 $$\upomega =\upmu ^{{2}} {/2}\upeta$$

**Table 1:** Electronegativity values (*X*), hardness (*η*), softness (*S*) and electrophysical (*ω*), chemical potential (*μ*), HOMO or LUMO orbitals energy, energy gap and bipolar moments for C_60_, [C_60_ + NO], and NO and energy formation (*E*_*F*_) of [C_60_ + NO] complex.

Compounds	E_HOMO_ [eV]	E_LUMO_ [eV]	ΔE_gap_ [eV]	μ [eV]	η [eV]	ω [eV]	S [eV]	X [eV]	E_F_ [kcal.mol^**−**1^]	Dipole moment [D]
C_60_	− 6.05	− 3.14	2.91	4.59	1.45	7.26	0.69	− 4.59	–	0
C_60_ + NO	− 8.94	− 7.64	1.30	8.29	0.65	52.86	1.54	− 8.29	5.16	12.92
NO	− 25.06	− 13.67	11.39	19.36	5.69	32.91	0.18	− 19.36	–	0.43

The energy levels involved in forming the nanocomplex and predicting its activity and performance play a crucial role in determining the effectiveness against the mass of cancer cells. Factors such as the hardness and softness values, along with the surface electronegativity of both the nanocomplex and the tumor, are important considerations. Simply put, the energy involved in creating the nanocomplex and predicting its effectiveness is essential to identify cancerous masses. Other important parameters obtained in the base state include the Mulliken charge, the complex dipole moment, and the electron localization function (ELF). ELF analysis offers reliable insight into the exchange within virtual and filled orbitals. Enhanced charge delocalization from NO orbitals to fullerene virtual orbitals indicates increased conjugation, which contributes to the stability of the complex^49^. The size of the bipolar moment is a very important parameter, which along with the electrostatic potential (ESP) represents the solubility of the complex^50^.

Using the components of each optically modified structure (NO, C_60_ and [C_60_ + NO] complex) and using the generalized Reduced Density Gradient (RDG) method, which is a convenient visual method for examining weak bonds such as van der Waals, the overbite overlay space is well specified. Using the Charge Decomposition Analysis (CDA) method, the cleavage of the ligand field was well established for the [C_60_ + NO] complex. These data were fully consistent with the Density of State (DOS) data that will be discussed below^51^.

One of the most important parameters for the determination of the surface charge in spherical systems is the use of the Radial Distribution Function (RDF). This electron charge analysis offers a very interesting comparison of the internal and external surface charges of spherical systems such as fullerene C_60_ based on the defined radius^52,53^. After optimizing the structure of the [C_60_ + NO] complex, the Natural Bond Orbital (NBO) analysis is performed using the same method and the previous set of assemblies, namely B3LYP/6–31 + G*, and the results are calculated and extracted purely for the electron transfer between NO and C_60_ in the complex. NBO provides an analysis of multi-electron wave functions in pairs of transitions. The NBO method uses wave functions from the first-order matrix of the reduced density. Therefore, this method is applicable to the general mathematical forms of wave functions. Using this method, it is possible to calculate the electron transmissions in different types of interconnections and links and obtain a suitable justification for other parameters^54,55^. Since fullerene C_60_ has the ability to accept 6 electrons, it is commonly used in donor–acceptor systems such as solar cells and the like. However, in the current study system due to the low orbital energy of LUMO NO, fullerene is the donor.

### Excited state details

Excited state calculations are performed in order to investigate the optical properties of the [C_60_ + NO] complex, using the TD-DFT computational method^56^. These calculations are carried out to obtain absorption spectra, distribution and determination of charge transitions in this complex using the CAM-B3LYP method and the base set of 6–31 + G* assemblies. The CAM-B3LYP method that has been modified is the B3LYP method in the excitation mode^57^. The primary distinction between B3LYP and CAM-B3LYP functionals lies in the inclusion of exact Hartree–Fock (HF) exchange. CAM-B3LYP integrates the hybrid characteristics of B3LYP. Atomization energies obtained from CAM-B3LYP are comparable in quality to those from B3LYP. Additionally, CAM-B3LYP exhibits strong performance in modeling charge transfer excitations in a dipeptide, an aspect that B3LYP significantly underestimates. It's crucial to note that the CAMY-B3LYP functional differs from B3LYP, as it employs a distinct switching function^58,59^. Charge transitions for the first five modes of excitation with the lowest energy have been performed based on the hole-electron theory^60^. Determining the wavelength of the CT and using the Marcus theory, the electron transfer rate is obtained between the two components of the complex. All DFT and TD-DFT calculations are performed using Gaussian 03, Revision C. 02 package^61^, and some analyzes are performed using Multiwfn 3.3.9^51^, VMD 1.9.2^62^, and GaussSum software^63^.

The Marcus theory is a common theory of electron transport in chemistry^64^. This theory has been widely accepted over the past few decades for making interesting predictions about the rate of electron transfer. Using the maximum wavelength for CT, applying the Planck equation and the Rehm-Weller equation, some of the other physical and chemical parameters such as the free energy of electron activation, the electron transfer rate between the two complexes, and the lifetime of the spectrometry comfort time have been calculated. These calculations have been performed with proper approximation and taking into account the lowest energy of charge transfer^65^.

## Results and discussion

### Structural analysis

In this section, the results of the DFT (ground state) and TD-DFT (excited state) calculations are considered. After optimizing the structure of the [C_60_ + NO] complex, these two components were placed at 1.92 Å from each other. The fullerene has a dipole moment with an intensity of 0 D in the neutral state and without subtraction. As shown in Fig. 1, the formation of its complex with NO greatly increased its dipole moment and reached about 12.92 D. These dipole moments are completely consistent with the values of the Mulliken charge, indicating that the formation of this complex has led to the elimination of the charge loading order on the fullerene C_60_.

**Figure 2:**
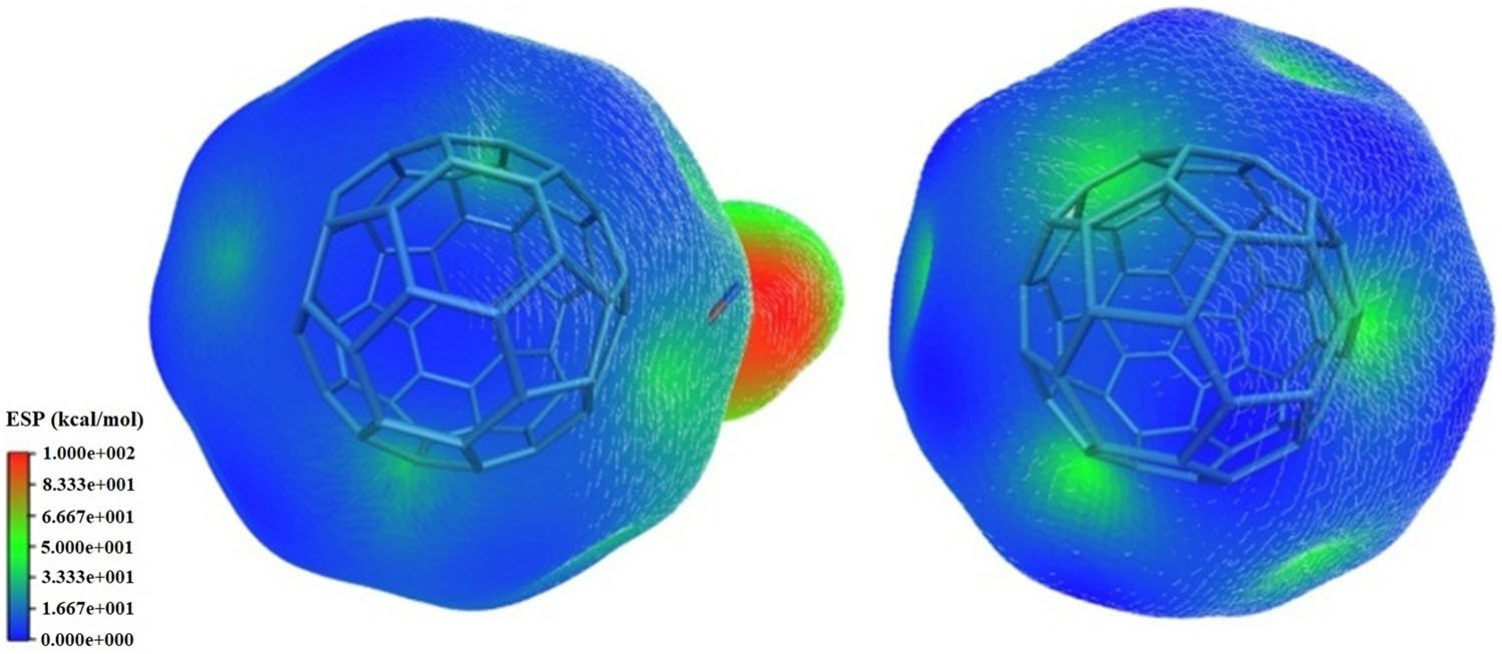
Electrostatic surface (ESP) images for complex [C_60_ + NO] and fullerene C_60_ in the gas phase calculated with the B3LYP/6–31 + G* method.

These extreme changes in the bipolar moment are easily understood using ESP (Fig. 2). The approach of NO to fullerene C_60_ resulted in electron turbulence induced from the NO side into a fully integrated fullerene system, such that the complex formed was completely bipolar and transformed into a complex with hydrophilic and hydrophobic ends. This feature appeared in the complex indicating its efficacy for drug delivery, especially in the passage of certain membranes and cellular barriers. ESP images can be used to predict the performance of such systems by simulation alone. Choosing a target molecule on the tumor cell and specifying its ESP through simulation, it is possible to design a drug carrier with a similar ESP for the purpose of intelligent drug delivery.

**Figure 3:**
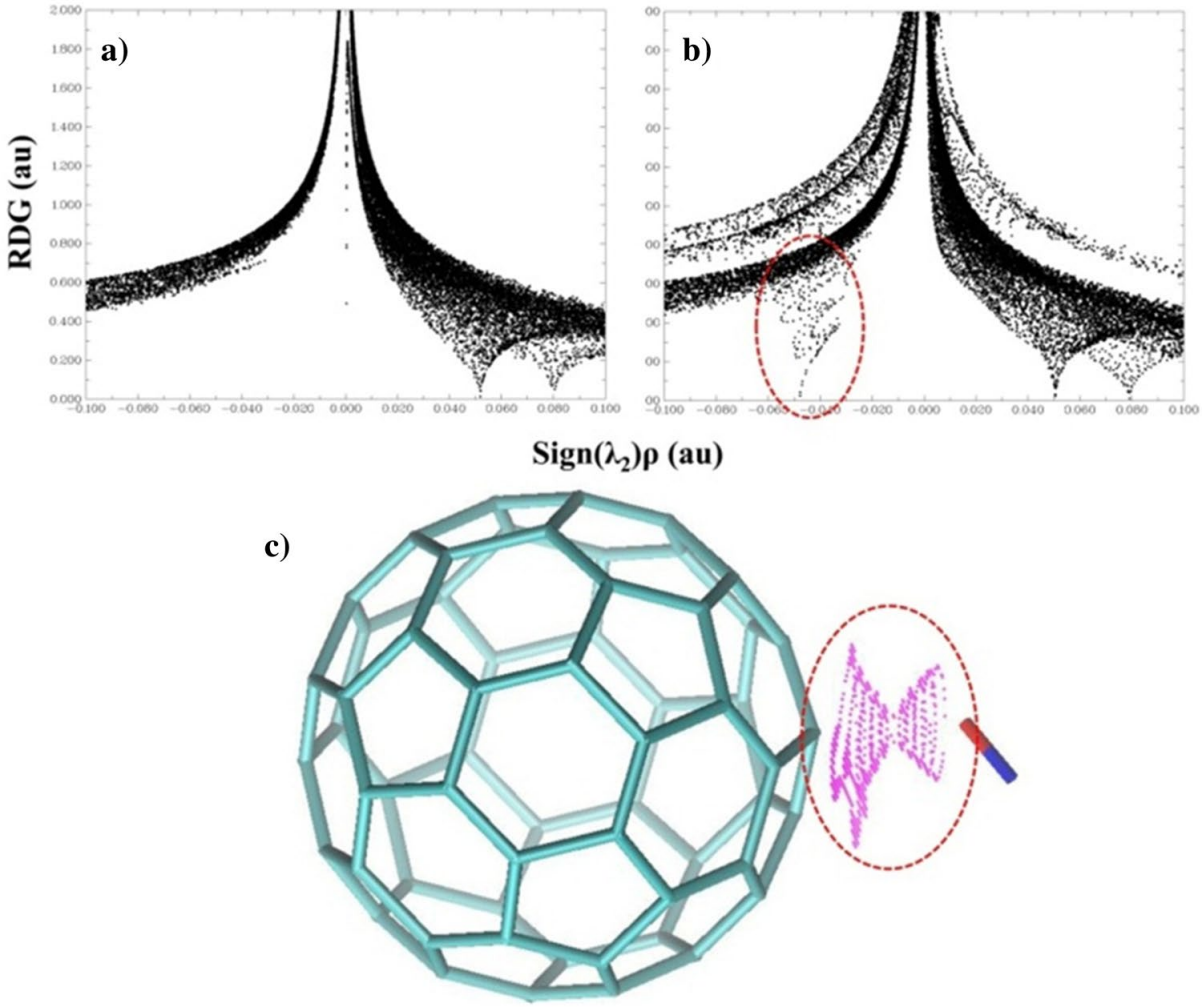
Investigating the links and interconnects (**a**) C_60_, (**b**) Determining the bonding and interconnects of the complex [C_60_ + NO] with the drawing of RDG/*sign(λ*_*2*_*)ρ*, (**c**) orbital overlap between NO and fullerene C_60_ in the [C_60_ + NO] complex.

During complex formation, the NO molecule moves to a position 1.92 Å away from the fullerene molecule, creating a weak interconnect between them. In Table 1, the formation energy of a complex based on the Gibbs free energy is *E*_*F*_ = 5.16 kcal.mol^−1^. This weak interconnect can be seen in Fig. 3 based on graphical data of RDG analysis. Figure 3a,b correspond to the C_60_ and [C_60_ + NO] complex, which have been achieved with drawing RDG on *sign(λ*_*2*_*)ρ*. *Ρ* is the electron density at *r* distance, which shows the critical points in chemical bonds and is very useful for checking interconnections in such complexes. As shown in these images, solid chemical bonds for C_60_ appeared in the x-axis at 0.05 and 0.08, and a weak peak appeared in the region of − 0.05 after the formation of the complex that is related to the interconnection between the two forming complex species. This peak is clearly visible in Fig. 3c as an orbital overlap between the two components of the complex. The drug carrier's polarity, coupled with a complex formation energy lower than 6 kcal·mol^−1^, highlights its potential for delivering drugs or other biocompounds. This dual advantage involves selective delivery based on polarity and efficient delivery by expending a minimal amount of energy.

**Figure 4:**
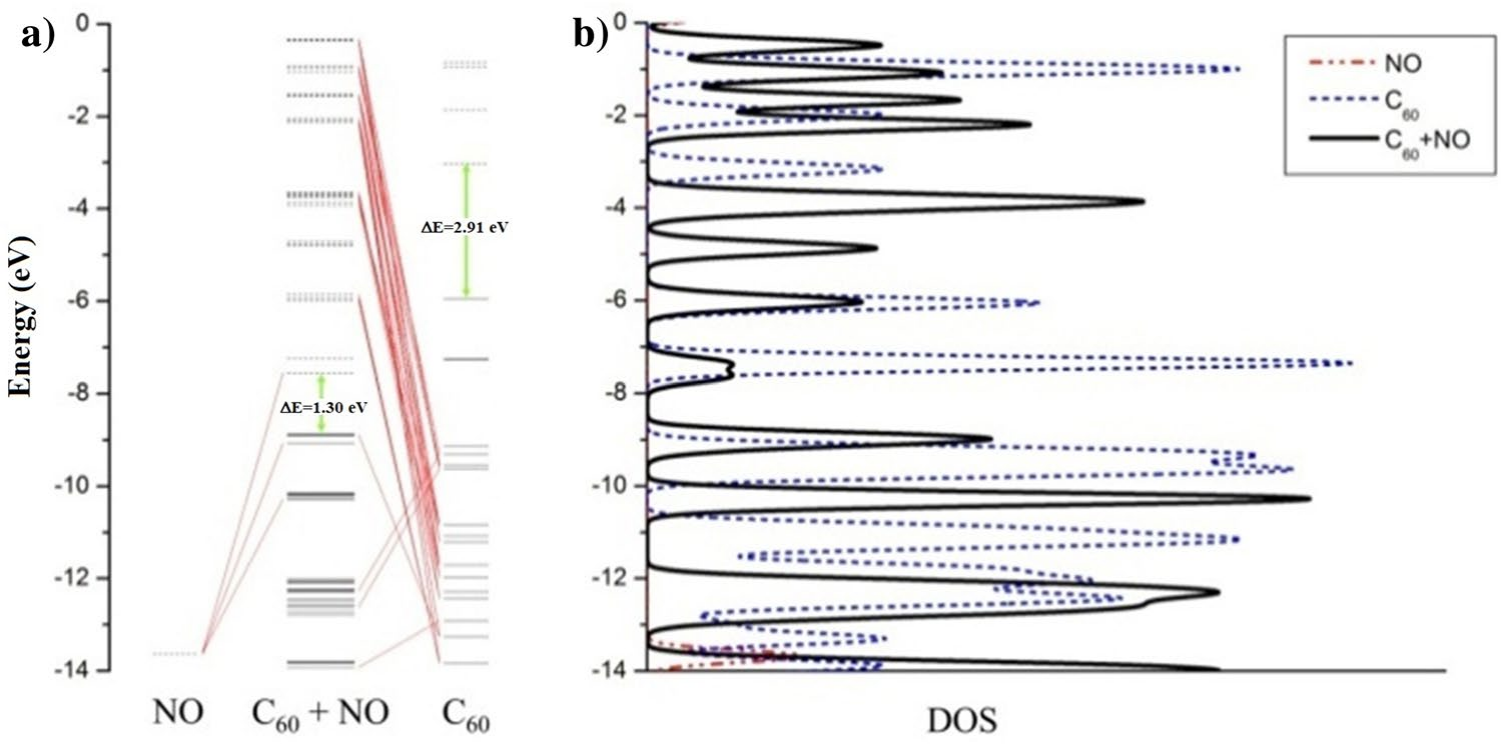
(**a**) Charge decomposition analyses (CDA), (**b**) density of state (DOS) for NO, fullerene C_60_ and [C_60_ + NO] complex in the range of − 14 to 0 eV.

The use of the CDA method provides a suitable approximation of the orbital levels in the [C_60_ + NO] complex. The CDA in Fig. 4a shows how the orbital NO levels reduce the energy level of the HOMO or LUMO fullerene orbitals. In addition, the formation of this complex caused the C_60_ energy gap drop from 2.91 eV to 1.30 eV. The CDA is based on an orbital fragment that exhibits molecular orbitals in isolated state. This method can be used to represent orbital alignments in complexes. As shown in Fig. 4b, the CDA results are completely consistent with the DOS results. Of course, it should be noted that according to the approximation used in the CDA drawing, the number and type of participation of each level in the complex is changed. To avoid confusion, only the range of − 14 to 0 eV is plotted in the Fig. 4, and only the participation of the levels with a high proportion have been considered. As shown in Fig. 4b, the fullerene energy gap in the complex is reduced to 1.61 eV, all of which are given in Table 1.

According to the results of Table 1, after the formation of the [C_60_ + NO] complex, the HOMO C_60_ orbital level is reduced to 2.82 eV, which is 4.5 eV for the LUMO orbital. The main reason for this decrease in the energy level of HOMO or LUMO orbitals is the low energy level of these orbitals in NO gas, which after overlapping strongly affects the energy levels and the gap. In Fig. 4a, it is also obvious the participation of the highest antioxidant NO bond with LUMO and HOMO orbitals in the complex. Using the energy of orbital HOMO and LUMO, in addition to obtaining the energy gap, valuable data can be obtained, among which the most important are electronegativity (X), hardness (η), softness (S), electrophysical (ω) and chemical potential (μ).

**Figure 5:**
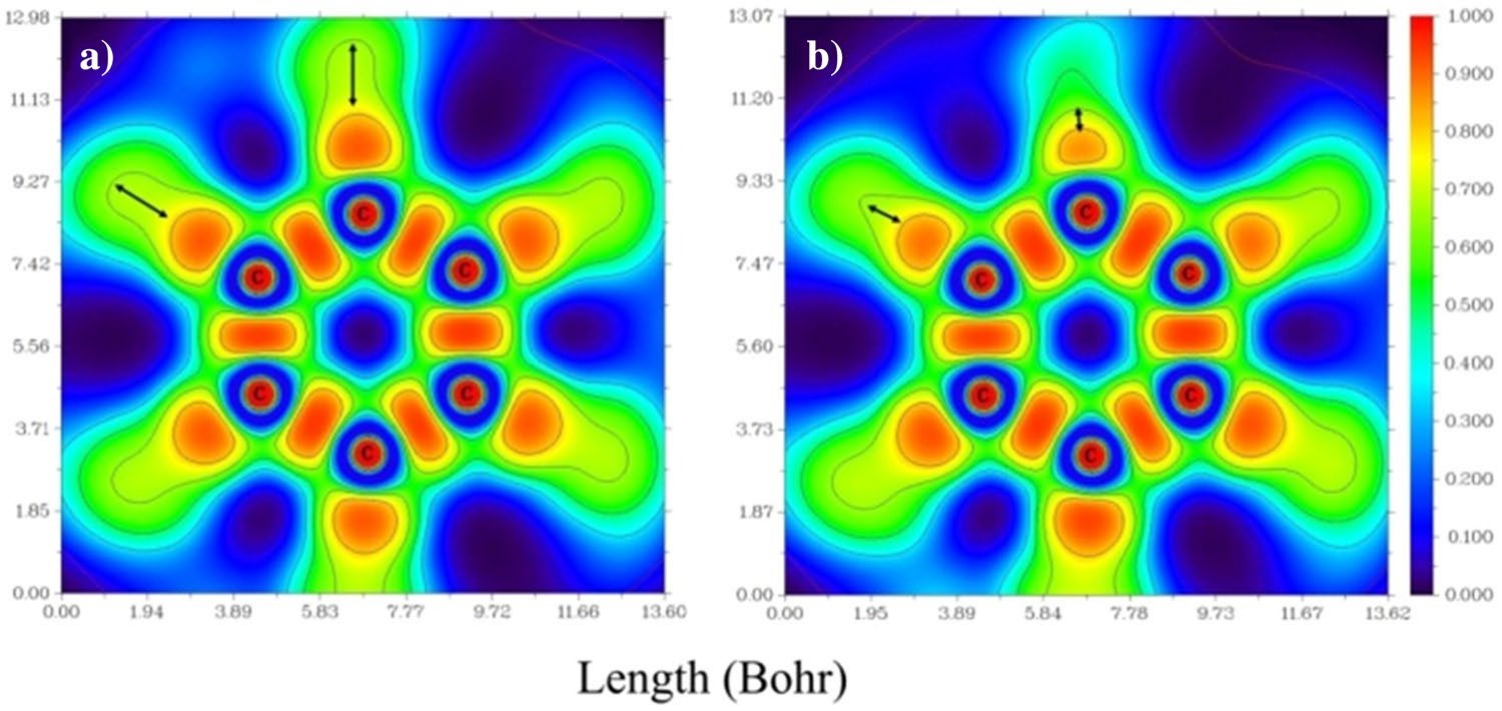
Electron density (**a**) ELF, one of the six-membered loops of fullerene C_60_ and (**b**) six-membered loops of fullerene C_60_ in the [C_60_ + NO] complex.

The study of hardness and softness in drug release systems is important for the reaction of hard type with hard species and the reaction of soft type with soft species. Therefore, it can be designed and synthesized with simulating the goal of an optimal drug release system. Other results from Table 1 include electronegativity and chemical potentials. The higher the electronegativity in a structure, the more stable it is. Therefore, the C_60_, [C_60_ + NO] and NO are the most stable, respectively. Unlike the electronegativity, the chemical potential indicates the chemical reactivity and its value is completely inverse to the electronegativity, which is presented in Eq. (2) in the methodology. Therefore, NO gas is recognized as the most reactive and unstable structure in Table 1. In fact, this reactivity is high for NO, which led to the formation of the [C_60_ + NO] complex. The formation energy of the complex is *E*_*F*_ = 5.16 kcal.mol^−1^ and represents a weak interconnect between the two species. The dipole moment formed in 12.92 D is also calculated for the complex based on molecular charge calculations. This large dipole moment for the complex can be understood by looking at Fig. 1 again. A very large dipole between NO oxygen molecule and one of the opposite carbons in the fullerene C_60_ has been created. The effect of this dipole is quite evident in Fig. 5. The ELF, which in fact represents the probability of the presence of electrons in the neighborhood of reference electrons with the same spin, separates the electrons of the valence layer from the nuclei and visualizes the electron properties of the chemical systems. By comparing Fig. 5a,b, the ELF of the six-membered fullerene C_60_ in its original state and after the formation of the [C_60_ + NO] complex was investigated. The presence of NO in the proximity of fullerene C_60_ changes its electronic structure, leading to a significant decrease in the electron density on the surrounding carbon atoms. This change is shown visually in Fig. 5 and is consistent with the Mulliken charge results.

In the analysis of the NBO calculations, since the effect of non-quantitative deployment or the interaction between the donor–acceptor orbits accumulated, it is natural that they are presented as a donor–acceptor of the CT or the generalized Lewis acid type. In Table 2, the energy values of the CT in the [C_60_ + NO] complex and the internal transfers of C_60_ in the presence and absence of NO are presented. In the [C_60_ + NO] complex, the largest number of transfers is from the C_60_ → NO. Although the difference between these transfers is very close, the most CT from C_60_ → NO is due to σC_49_-C_50_ → π*N–O and σC_50_-C_53_ → π*N–O with an energy equal to 0.45 kcal.mol^−1^ from the transfer type σ → π*. In the NO → C_60_ transfers, the largest transmission is related to LP O → σ*C_37_-C_50_ with an energy of 0.13 kcal.mol^−1^. This charge exchange between C_60_ and NO has changed the integrated C_60_ electron system. For example, the transfer of σC_49_-C_50_ → σ* C_48_-C_49_ from 2.89 to 2.82 for C_60_ dropped. The most changes in the amount of energy was associated with the transitions π → σ* and π → π*, which had a progressive and decreasing trend after the formation of the [C_60_ + NO] complex, respectively. Among these, πC_48_-C_49_ → σ* C_47_-C_48_ can be mentioned, which after the formation of the complex increased from 1.35 to 13.80 kcal.mol^−1^ and the transfer of πC_48_-C_49_ → π* C_47_-C_57_ from 13.05 to 1.47 kcal.mol^−1^ dropped. A striking point in Table 2 is the loss of the π bond on the C_50_ atom and the creation of a LP on it. This irregularity in load transitions is fully justified by the increase in bipolar moment and electron density observed in the previous sections.

**Table 2:** NBO calculated donor and acceptor orbital energies for C_60_ and [C_60_ + NO] complex with kcal.mol^**−**1^ unit.

NO → C_60_ & C_60_ → NO	E	C_60_ → C_60_	E C_60_	E [C_60_ + NO]
σN-O → σ*C_49_-C_50_	0.07	σC_49_-C_50_** → **σ* C_37_-C_38_	3.27	3.25
σN-O → σ*C_50_-C_53_	0.07	σC_49_-C_50_** → **σ* C_37_-C_50_	1.75	2.82
σN-O → π*C_48_-C_49_	0.07	σC_49_-C_50_** → **σ* C_47_-C_48_	2.99	3.25
σN-O → π*C_52_-C_53_	0.07	σC_49_-C_50_** → **σ* C_48_-C_49_	2.89	2.82
πN-O → σ*C_49_-C_50_	0.11	σC_49_-C_50_** → **σ* C_49_-C_55_	1.14	1.16
πN-O → σ*C_50_-C_53_	0.11	σC_49_-C_50_** → **σ* C_50_-C_53_	0.94	1.16
LP O → σ*C_37_-C_50_	0.13	σC_49_-C_50_** → **σ* C_52_-C_53_	2.94	2.77
LP C_50_ → π*N–O	0.68	σC_49_-C_50_** → **σ* C_55_-C_56_	2.66	2.77
σC_37_-C_50_ → π*N–O	0.31	πC_48_-C_49_** → **π* C_36_-C_62_	14.29	13.80
πC_38_-C_51_ → π*N–O	0.07	πC_48_-C_49_** → **σ* C_47_-C_48_	1.35	13.80
πC_48_-C_49_ → σ*N–O	0.05	πC_48_-C_49_** → **π* C_47_-C_57_	13.05	1.47
σC_49_-C_50_ → π*N–O	0.45	πC_48_-C_49_** → **σ* C_49_-C_50_	1.17	13.80
σC_50_-C_53_ → π*N–O	0.45	πC_48_-C_49_** → **σ* C_49_-C_55_	1.49	1.47
πC_52_-C_53_ → σ*N–O	0.05	πC_48_-C_49_** → **π* C_55_-C_56_	14.19	1.47

**Figure 6:**
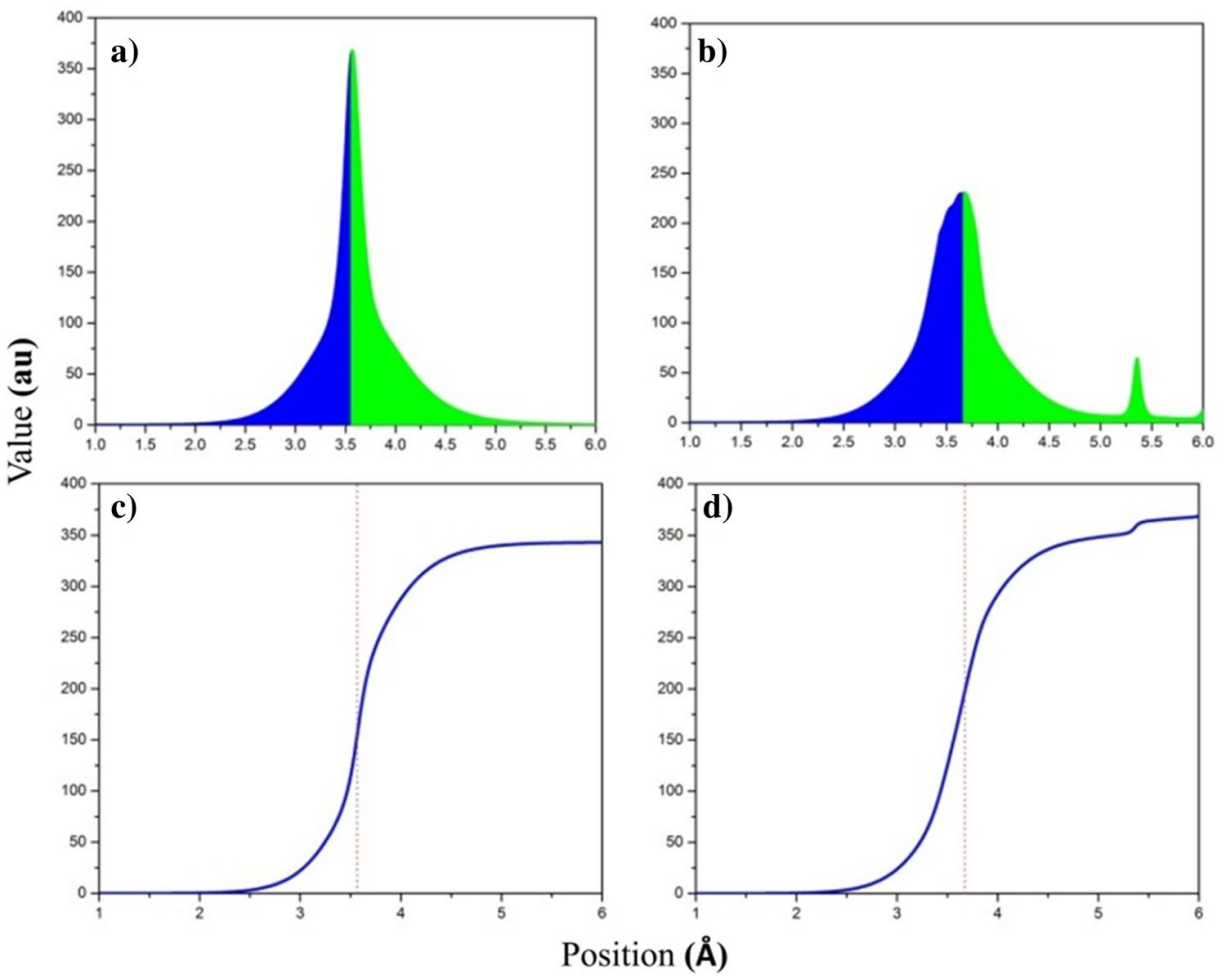
Radial distribution function for (**a**) fullerene C_60_ and (**b**) [C_60_ + NO] complex and integration curve of RDF for (**c**) fullerene C_60_ and (**d**) [C_60_ + NO] complex.

The charge distribution in fullerenes such as the C_60_ or equivalent clusters is usually externally slightly above the inner surface due to the orientation of their orbital π in the spherical system. Using the RDF analysis, the C_60_ radius is compared and studied in the complex, as shown in Fig. 6. Figure 6a,b correspond to the fullerene C_60_ and the [C_60_ + NO] complex, respectively. The green parts correspond to the external surface charge distribution and the blue part to the internal surface charge. In Fig. 6a, the charge distribution on the outer surface is slightly larger than that on the inner surface. Figure 6c,d are actually integral curve under the surface of Fig. 6a,b. The internal and external charge space is actually the same as that of the sphere studied, which for C_60_ alone and C_60_ in the [C_60_ + NO] complex at the values of 3.56 and 3.67 Å was obtained. The value on the x-axis is in fact the density of the electron charge around the nuclei, expressed as the energy necessary to cross the sphere wall. Comparing the distribution of the load, it can easily be seen that the fullerene radius in the complex is slightly larger than normal and the load distribution on the inner surface is slightly larger, which was predictable from the NBO calculations. However, the NO surface charge contribution alone should not be taken at a radius of 5.37 Å, which has a peak so that its share of the C_60_ alone can be determined.

The increase in the ratio of internal to external charge distribution can be attributed to two major factors. First, the C_60_ → NO charge transmissions are much higher than the NO → C_60_ and these transfers are from the external level of the C_60_. Second, the loss of a π bond and transformation to LP has reduced the pressure of the inner lops of the π orbital and is more than superficial charge pushed into, and even the formed LP may have been oriented toward fullerene. Another point of the Fig. 6 analysis is that the amount of energy needed to navigate the fullerene wall after the formation of a complex has dropped from a range of 350 (au) to less than 250 (au), which is of course consistent with increase in radius of C_60_ and decrease in charge density around the nucleus. For a closer look, the ELF electron density on Fig. 5 can be referenced.

### Optical analysis

**Figure 7:**
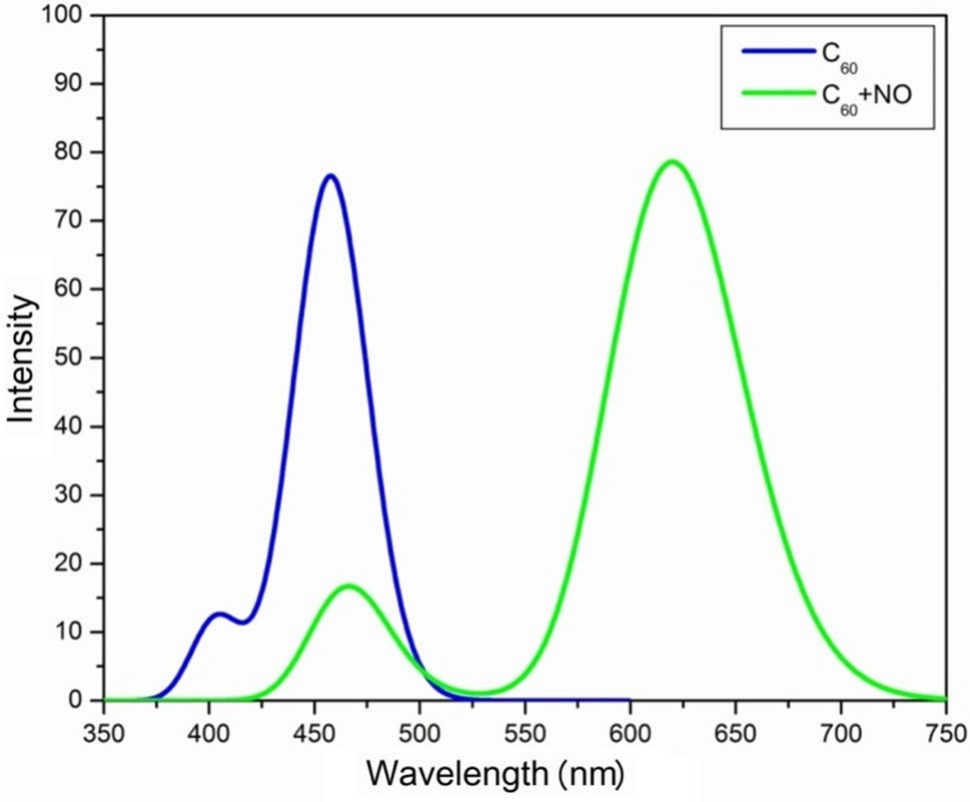
UV–Vis absorption spectrum for fullerene C_60_ and [C_60_ + NO] complex.

Calculating the excited state, the optical absorption spectra of fullerene C_60_ and [C_60_ + NO] complex is calculated in the gas phase. Figure 7 shows that after the formation of [C_60_ + NO], the absorption spectrum of a large red shift is about 200 nm. This displacement in absorption wavelengths is due to the increase in the energy levels of HOMO and LUMO orbitals, as previously presented in Fig. 4. The energy levels of HOMO or LUMO orbitals for fullerene C_60_ are about − 6 and − 3 eV, respectively, and these values for the [C_60_ + NO] complex are about − 9 and − 8.5 eV, respectively. This energy reduction on the orbital surface has led to excitation at higher wavelengths with less energy, resulting in a large red shift in the maximum absorption wavelength.

**Figure 8:**
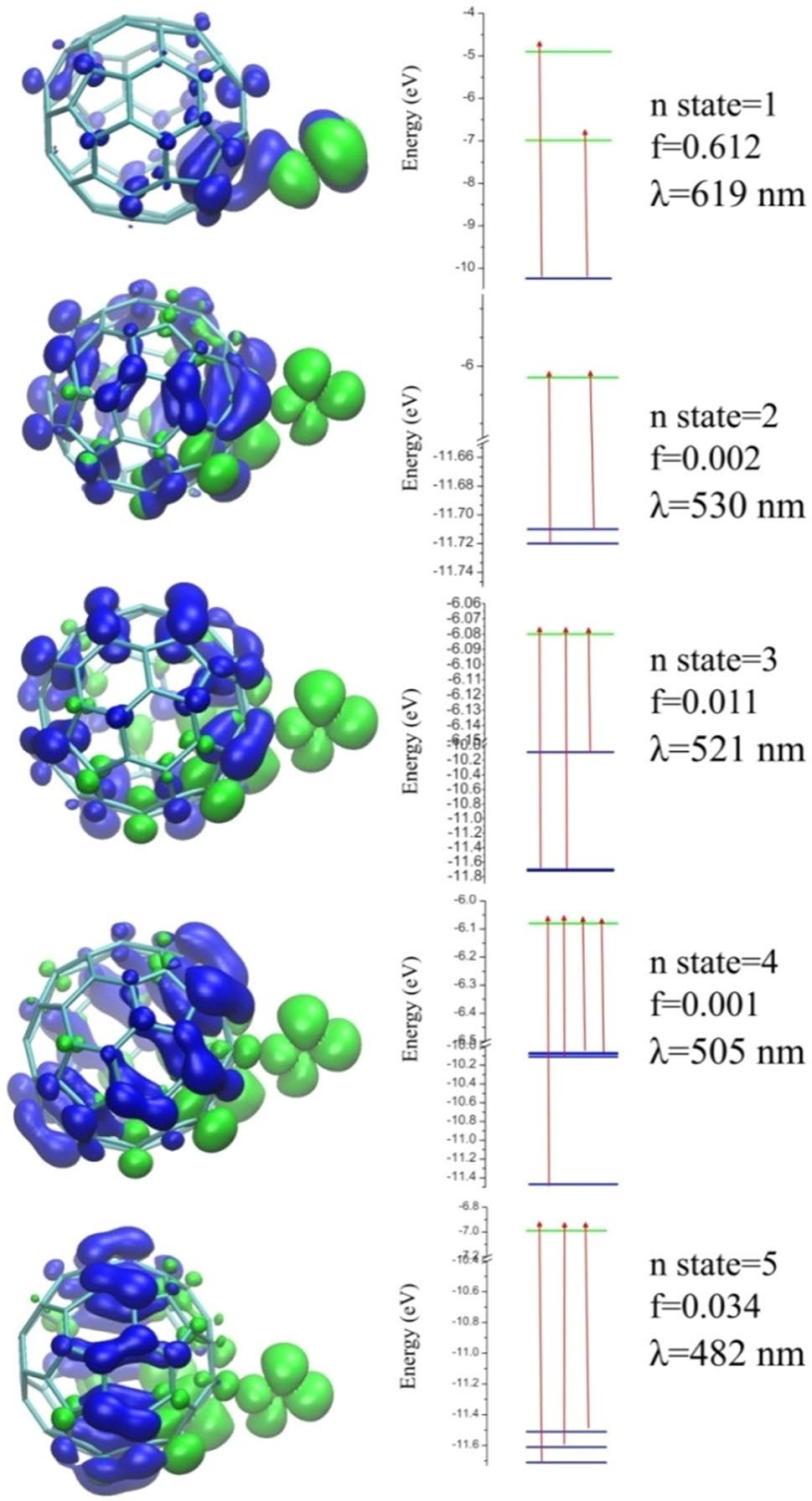
Orbitals for hole and electron in the PET process for complex [C_60_ + NO].

The results of electron transitions in the excited state for the first five states are presented in the Fig. 8. According to these results, the photo-induced electron transfer (PET) process has occurred for almost every first five cases of arousal. The significance of this process is that in the first five modes of excitation, the least amount of energy is needed to transmit electrons. Typically, if the PET process is from the chelator to the fluorophore, it will turn off the fluorescence emission spectrum. In Figs. 7 and 8, it is clear that in the [C_60_ + NO] complex the first mode of excitement occurs by absorbing wavelengths at 619 nm. This largest absorption peak is about *f* = 0.612 and is much higher than the other four. In Fig. 8, green orbitals play a role of electron and blue orbitals play the role of a hole. The greater the difference between these two types of orbitals, the more likely it is for PCT and PET. The highest resolution of arousal was done at *nstate* = 5 at 482 nm. Also, in this form, the electron transmission related to the levels for each absorption has been specified. PCT and PET have a direct impact on the fluorescence spectra, a well-recognized method for drug carrier detection. The observation of a red shift in the UV–Vis spectrum indicates inevitable changes in fluorescence, which serves as a favorable factor for detection^66^.

Using the Marcus theory, the electron transfer rate in donor–acceptor systems such as [C_60_ + NO] with an appropriate approximation can be calculated. The data are presented in Table 3. Applying Eq. (7), it is possible to calculate the free energy of electron transfer. Since in each of the first five modes, the excitement of the PET process is evident, the absorbed wavelength can be considered equal to *λ*_*max*_ of the electron transfer. The Eq. (7) is energy–wavelength relation, which relates the energy change (*∆E*) of a photon to its wavelength (*λ*), where *h* is the Planck constant, and *c* is the speed of light.

7 $$\Delta {\text{G}}_{{{\text{et}}}}^{\# } = \Delta {\text{E}} = {\text{hc/}}\uplambda$$

**Table 3:** The calculated data (*ΔG*_*et*_, *ΔG*^*#*^_*et*_, *ΔE*_*Ox-Red*_, *k*_*et*_ and *λ*_*et*_) of the electron transfer and/or PET-process between NO and fullerene C_60_ molecules.

nstate	λ_et_ [nm]	ΔG^#^_et_ [kcal mol^**−**1^]	k_et_ [m s^**−**1^]	ΔG_et_ [kcal mol^**−**1^]	ΔE_Ox-Red_ [V]
1	619	46.16	8.08*10^−22^	59.80	2.62
2	530	53.91	1.67*10^−27^	80.73	3.53
3	521	54.84	3.48*10^−28^	83.46	3.65
4	505	56.58	1.84*10^−29^	88.70	3.88
5	482	59.28	1.92*10^−31^	97.14	4.24

Obtaining the values of the free energy activating the electron transfer *ΔG*^*#*^_*et*_, the values of electron transfer velocity *k*_*et*_ can be calculated using Eq. (8). To find the value of the Gibbs free energy change (*∆G*_*et*_) from the Gibbs free energy of activation *ΔG*^*#*^_*et*_, Eq. 9 is employed. The value *L* is the reorganization energy, parameter related to the energy needed to reorganize the surrounding environment during the reaction.

8 $${\text{k}}_{{{\text{et}}}} = {\text{k}}_{0} \exp \left( { - \Delta {\text{G}}_{{{\text{et}}}}^{\# } {\text{/R}}} \right)$$

9 $$\Delta {\text{G}}_{{{\text{et}}}}^{\# } = \left( {{\text{L}}/4} \right) \, \left( {1 + \Delta {\text{G}}_{{{\text{et}}}} {\text{/L}}} \right)^{2}$$

Finally, the electrodes potential difference in the calculation conditions, namely in the gas phase, can be estimated using the Eq. (10) (Rehm-Weller) and through the energy values of free electron transfer.

10 $$\Delta {\text{G}}_{{{\text{et}}}} = {\text{e}}\left[ {{\text{E}}_{{\text{D}}}^{{\text{o}}} - {\text{E}}_{{\text{A}}}^{{\text{o}}} } \right] - \Delta {\text{E}}^{*} +\upomega _{1}$$

The Eq. (10) predicts the free energy variations between an electron donor and an electron acceptor. In this equation, *e* is the unit electric charge, *E°*_*D*_ and *E°*_*A*_ are the reduction potentials of the electron donor and electron acceptor, respectively, ΔE* is the energy of the single and triple excited states, and *ω*_*1*_ is the input required for the donor and acceptor within the range of the electron transfer distance.

According to the results obtained in Table 3, the highest electron transfer rate associated with excitation is in the first state, which requires the least amount of energy to carry out this electron transfer with a velocity equal to 8.08*10^−22^ m·s^−1^. According to the calculations made in the gas phase, this electron transfer can be accomplished by applying potentials up to 2.62 V (difference between the reduced and oxidized electrodes). Thus, with the help of the wavelength of arousal, it is possible to predict the potential difference between the two species to carry out the electron transfer process with an appropriate approximation. Table 3 contains additional findings for the remaining four excitation modes, showing the reduced electron transfer rate between C_**60**_ and NO as the absorption wavelength decreases.

## Conclusion

In this research, the design and properties of the new conjugated fullerene C_60_ molecule with nitric oxide were verified using computational methods. The calculated values show a promising result for the use of [C_60_ + NO] complex in different fields of biomedicine. The optimized structure of the [C_60_ + NO] complex has special electrical and optical properties. According to the molecular charge calculations using the DFT method, the formation of the C_60_ complex with NO was a highly polar complex with a bipolar moment of about 12.92 D. Also, the calculated ESP showed well the changes in the bipolar moment between the C_60_ and the [C_60_ + NO] complex. The formation of this complex reduced the energy level of the HOMO or LUMO complex orbitals, which was reflected in an increase of the reactivity of the [C_60_ + NO] complex with the fullerene C_60_.

The NBO calculations showed a partial charge transition between the two constituents of the formed complex. The CT and the overlapping of orbitals caused the loss of one of the π orbitals of the fullerene in the vicinity of NO. The RDF analysis showed increased both fullerene radius due to the charge received from NO and the ratio of internal charge to surface charge due to the loss of one links of π orbital. The RDG analysis well described the orbital overlap between the two parts of the complex, which was in perfect agreement with the DOS results. The formation of the complex compared to the free fullerene C_60_ molecule not only reduced the energy level of the HOMO or LUMO orbitals, but also reduced the energy gap.

According to TD-DFT calculations, the absorption energy of the UV–Vis spectrum started at the complex with wavelengths higher than C_60_, causing a red shift of the absorbed wavelength. Using this template could be a great help in identifying the fullerene path before and after the possible reaction in the body. From the electron–hole theory data, five PET states were found in the first five modes of arousal, which determined the rate of this electron transfer using Marcus theory with an appropriate approximation. With increasing electron transfer, the electron transfer energy and the redox potential increase, while the wavelength and the constant of electron transfer velocity decrease.

The use of this type of calculation gives a very good insight into the understanding of light-sensitive donor–acceptor systems. The studied complex represents a promising molecule for diverse biological applications in medicine, with its unique properties offering opportunities for targeted drug delivery, imaging, therapy, and other innovative approaches. Further research should focus on the effect of the complex on tissue cultures and subsequently on trials with experimental animals in order to translate this interesting combination of two molecules into practical biomedical applications beneficial in various fields of medicine and improving human health.

## Data Availability

The data used and/or analysed during the current study are available from the corresponding author on reasonable request.
